# Possible impact of rising sea levels on vector-borne infectious diseases

**DOI:** 10.1186/1471-2334-11-18

**Published:** 2011-01-18

**Authors:** Ranjan Ramasamy, Sinnathamby N Surendran

**Affiliations:** 1 PAPSRB Institute of Health Sciences, Universiti Brunei Darussalam, Gadong, Brunei Darussalam; 2 Department of Zoology, University of Jaffna, Jaffna, Sri Lanka

## Abstract

**Background:**

Vector-borne infectious diseases are a significant cause of human and animal mortality and morbidity. Modeling studies predict that changes in climate that accompany global warming will alter the transmission risk of many vector-borne infectious diseases in different parts of the world. Global warming will also raise sea levels, which will lead to an increase in saline and brackish water bodies in coastal areas. The potential impact of rising sea levels, as opposed to climate change, on the prevalence of vector-borne infectious diseases has hitherto been unrecognised.

**Presentation of the hypothesis:**

Mosquito species possessing salinity-tolerant larvae and pupae, and capable of transmitting arboviruses and parasites are found in many parts of the world. An expansion of brackish and saline water bodies in coastal areas, associated with rising sea levels, can increase densities of salinity-tolerant vector mosquitoes and lead to the adaptation of freshwater vectors to breed in brackish and saline waters. The breeding of non-mosquito vectors may also be influenced by salinity changes in coastal habitats. Higher vector densities can increase transmission of vector-borne infectious diseases in coastal localities, which can then spread to other areas.

**Testing the hypothesis:**

The demonstration of increases in vector populations and disease prevalence that is related to an expansion of brackish/saline water bodies in coastal areas will provide the necessary supportive evidence. However the implementation of specific vector and disease control measures to counter the threat will confound the expected findings.

**Implications of the hypothesis:**

Rising sea levels can act synergistically with climate change and then interact in a complex manner with other environmental and socio-economic factors to generate a greater potential for the transmission of vector-borne infectious diseases. The resulting health impacts are likely to be particularly significant in resource-poor countries in the tropics and semi-tropics. Some measures to meet this threat are outlined.

## Background

Vector-borne infectious diseases (VBD) are a significant cause of morbidity and mortality in humans and animals. Important vectors of human VBD are closely associated with water bodies. They include mosquitoes that lay eggs in water to produce larvae, which feed and transform into pupae in water. Mosquitoes transmit many VBD including malaria, lymphatic filariasis and dengue with recently estimated prevalence of 247, 120 and 50 million cases worldwide respectively [1-3]. Schistosomiasis transmitted by snail vectors accounts for 207 million cases worldwide [4]. Snails become infected by miracidia produced from schistosome eggs in water and subsequently release cercariae that infect humans in water. The reduction of vector breeding and survival, and human-vector contact are important components in worldwide efforts to reduce the incidence of VBD.

Climate change due to global warming [5] can adversely affect human health [6-8]. Possible changes in the prevalence of mosquito VBD such as malaria and dengue as a result of global climate change have attracted particular attention [6-12]. Models forecast a generally increased risk of malaria transmission at greater latitudes in the present temperate zones and higher altitudes in tropical countries, although there is debate about the extent of the spread, and reduced transmission may occur in some areas [10-12]. Such predictions are based on the effects of changing temperature, rainfall and humidity on mosquito breeding and survival, and the more rapid development of ingested gametocytes to infective sporozoites in mosquitoes at moderately higher ambient temperatures. The predicted spread of mosquito vectors is supported by a discernible trend in the distribution of animals and plants attributed to global warming [13].

Global warming is expected to raise sea levels by 18 to 59 cm by the end of the 21^st ^century through the melting of glaciers and polar ice plus the thermal expansion of seawater [5,14]. Rising sea levels will affect the extent of saline (>30 parts per thousand or ppt salt) or brackish (0.5-30 ppt salt) water bodies in coastal areas. These include coastal estuaries, lagoons, marshes and mangroves [14]. Models suggest that the salinity of estuarine systems will rise and their boundaries move further inland with more pronounced tidal water flows into rivers [14]. A proportion of coastal wetlands such as salt marshes and mangroves will become inundated but additional saline wetlands will be formed further inland [14]. Rising sea levels, and higher water withdrawal rates from freshwater aquifers near the coast by expanding populations will increase saltwater intrusion in the aquifers [15]. These changes in turn will cause ponds, lakes and wells in coastal areas to become more brackish. Expansion of estuaries and lagoons heightens the risk of cholera transmission since *Vibrio cholerae *is associated with plankton in such waters [16]. However the possible impact of rising sea levels, as opposed to climate change, on the prevalence of VBD has hitherto been unrecognised [6-12]. This article proposes that an expansion of brackish and saline water bodies in coastal areas caused by rising sea levels can increase transmission of important VBD.

## Presentation of the hypothesis

### Some mosquito vectors possess physiological and structural adaptation to breed in brackish and saline waters

While most mosquito vectors breed in freshwater, some species that breed in brackish/saline waters are important vectors of human disease [[17], Additional file 1]. Their larvae possess cuticles that are less permeable to water than freshwater forms, and their pupae have thickened and sclerotized cuticles that are impermeable to water and ions [18]. Salinity-tolerant mosquito larvae possess varying osmoregulatory mechanisms e.g. *Aedes taeniorhyncus *drink surrounding fluid and excrete Na^+ ^and Cl^- ^from the posterior rectum to produce a hyperosmotic urine [18], *Culex tarsalis *larvae accumulate proline and trehalose in haemolymph to maintain iso-osmolarity in brackish waters [19], and *Anopheles albimanus *larvae differentially localize Na^+^K^+ ^ATPase in rectal cells in fresh or saline water for osmoregulation through ion excretion [20].

### Expansion of brackish and saline water bodies can lead to increased breeding of salinity-tolerant mosquitoes and greater disease transmission risk

The Asian tsunami of December 2004 provided relevant examples. *Anopheles sundaicus *s.l., a widespread malaria vector along Asian coasts [21,22] increased in density in the Andaman and Nicobar islands following the intrusion of sea water inland, concomitant with a rise in *Plasmodium falciparum *infections [23]. Increased densities of *Culex sitiens*, an established vector of arboviruses [17], and *An. sundaicus *s.l. were also observed in an area of Thailand affected by the tsunami [24].

The consequences of human-induced ecological changes provide another set of examples. Large-scale shrimp farming in the Mekong delta of Vietnam locally increased the density of *An. sundaicus *s.l. [25]. Aquaculture is an expanding economic activity along tropical Asian coasts. Also, higher densities of *Aedes (Ochlerotatus) camptorhynchus*, a vector of Ross River virus (RRV), have been associated with increasing salinisation of freshwater bodies due to intensive agriculture in Western Australia [[26-29], Additional file 2].

Conversely, a reduction in the habitat of a brackish water vector has historically been associated with a decrease in malaria in Western Europe. *Anopheles atroparvus *of the *An. maculipennis *complex was primarily responsible for transmitting malaria until the early 1900s in marshland areas of England [30] and low lying areas of Western Europe like the river deltas of the Netherlands [31]. The draining of coastal marshes, and the attendant reduction in breeding sites for *An. atroparvus*, contributed significantly towards eliminating malaria in these areas [30,31].

### Freshwater-breeding mosquito vectors can adapt to increased salinity with greater availability of brackish and saline water breeding sites

Increasing salinity in water bodies can reduce populations of mosquito vectors that are unable to tolerate salinity, thereby tending to reduce disease transmission. However, the greater availability of brackish water bodies can also lead to freshwater breeding mosquitoes adapting to breed in them e.g. typically freshwater mosquitoes *An. stephensi *and *An. culicifacies*, were found breeding in brackish water bodies immediately after the 2004 tsunami in India [32] and five years later in eastern Sri Lanka [33]. It is unclear whether the egg laying and larval development in brackish water seen in these instances involved reversible physiological adaptations [18-20] or genetic changes. Genetic selection for greater salinity tolerance in larvae has been observed in *Ae. taeniorhynchus *in the laboratory [18], and *Ae*. *camptorhynchus *in the field in Western Australia [28]. Cases of recent genetic selection for salinity tolerance have not been reported for anopheline vectors of malaria but the existence of sibling species that differ in salinity tolerance within anopheline species complexes such as Subpictus [34], Sundaicus [21,22], Gambiae [35] and Farauti [36] shows that this is clearly possible over a period of time.

There are no reports to date of *Aedes aegypti *or *Ae. albopictus*, the major vectors of dengue and other arboviruses, adapting to breed in brackish water. *Ae. aegypti *larvae possess the necessary physiological mechanisms to withstand a limited, short-term increase in salinity [37]. It is therefore possible that instances of its field adaptation to brackish/saline waters have gone unnoticed. *Cx. tarsalis*, the most important vector of arboviruses along the west coast of North America, however breeds in both fresh and brackish waters [38].

### Other human disease vectors may tolerate brackish waters or soil salinity

*Biomphalaria *and *Bulinus *species snails are intermediate hosts that transmit human schistosomiasis in freshwater. Other diseases caused by trematode flukes for which freshwater snails serve as intermediate hosts include fascioliasis, pragonimiasis and clonorchiasis. The impact of climate change, but not a rise in sea levels, on trematode VBD has recently been reviewed [39]. Construction of the Diama dam in the Senegal River basin in West Africa reduced tidal inflows and led to an increase in the prevalence of intestinal and urinary schistosomiasis that was at least partly ascribed to increased breeding of snail vectors after a reduction in water salinity [40,41]. On the other hand, observations in South America suggest that *Biomphalaria glabrata *and *Biomphalaria straminea *snail vectors can survive in water with 5 ppt salinity in coastal areas [42,43]. *Schistosoma mansoni *eggs have been reported to hatch in water with up to 6 ppt salinity [42]. Non-human, particularly bird-infective, schistosomes can however be transmitted in brackish water and are responsible for causing cercarial dermatitis or swimmer's itch.

Tsetse fly (*Glossina *spp) vectors of African trypanosomiasis are widespread in inland Africa. However *Glossina palpalis gambiensis *has also been reported to breed and transmit human trypanosomiasis in coastal locations in Guinea that are rich in mangrove swamps and where larval and pupal development takes place in soil [44]. Phlebotomine sandfly vectors of leishmaniasis that develop in a terrestrial habitat are found in coastal areas [45] but the ability of their eggs and larval stages to tolerate saline soils remains poorly studied. Their spread to increasing areas of brackish soils near coastal communities is a possibility.

### Increasing density of coastal vectors heightens the risk of disease transmission

An increased risk of disease transmission has been mathematically related to higher vector densities and other vector characteristics that can be influenced by climate change such as increased survival of vectors and a reduction in the extrinsic incubation period for pathogens [46].

Greater disease transmission in coastal areas can initially generate local foci of high transmission that can subsequently spread VBD to wider areas. The possible role of locally high densities of *An. annularis*, a minor freshwater vector, in initiating malaria epidemics in Sri Lanka is illustrative in this regard [[47], Additional file 2]. Efficient vectors that are able to breed or adapt to breed in both fresh and brackish waters, e.g. *An. culicifacies *[32,33] and *An. sundaicus *in Asia [21,22], and *Cx. tarsalis *in North America [38], can be particularly potent in disseminating VBD in this context.

It is difficult to predict a time-scale for the projected increased transmission risk of VBD since it will vary with the vector, pathogen and locality concerned. It will however probably parallel the expansion of coastal brackish/saline water bodies and be additionally influenced by changes in factors such as climate, agricultural practice, animal reservoirs of disease and human populations.

## Testing the hypothesis

Monitoring changes in relevant vector populations and disease incidence and prevalence near coastal brackish/saline water bodies in different parts of the world are needed to provide more substantial evidence. Since sea level rise and consequent expansion brackish and saline water bodies are gradual processes, the changes in vectors and disease patterns are also likely to be gradual, and require monitoring over a long period. However, additional measures undertaken to reduce vector populations and disease prevalence in coastal areas in light of the present hypothesis will diminish the predicted increased transmission of VBD.

Demonstrations of the ability of important mosquito vectors to tolerate and adapt to brackish/saline waters in the laboratory and in field situations will also help support the hypothesis. More research too on the ability of human schistosome eggs to hatch, miracidia to survive and infect snail vectors, longevity of snail hosts, and infectivity of cercariae released by snails in brackish water, and also the possibility of trematode parasites and their snail hosts adapting to brackish water in the long term is required. Relevant studies on the bionomics of non-mosquito vectors of African trypanosomiasis and other VBD are additionally needed. Also, little is presently known on how physiological changes associated with adapting to salinity might affect the vectorial capacity of mosquitoes and other vectors.

## Implications of the hypothesis

Modeling of the expected climate change in England predicts a greater potential for endogenous transmission of malaria [48]. However, this model did not consider the likely increased breeding of *An. atroparvus *in England resulting from rising sea levels causing an expansion of coastal marshland. Although an increased risk of malaria has to be planned for in the UK, it is unlikely that the disease will re-establish itself in the UK or other developed countries for a variety of reasons. These include good health services and socio-economic conditions, and a low and more seasonal transmission capacity due to the climate. However, the situation is different in many resource-poor countries in tropical and semi-tropical parts of the world. Because of a climate that is more conducive to the transmission of VBD and weaker health and social infrastructures, greater transmission in coastal areas can be expected to significantly increase the burden of malaria and other mosquito-borne diseases in such countries.

Licensed vaccines are presently available for only two arboviral diseases viz. yellow fever and Japanese encephalitis. Development of a vaccine against dengue is hampered by the existence of four virus serotypes and because a sub-optimal primary immune response can exacerbate disease caused by a subsequent infection [49,50]. Only drugs that provide symptomatic relief are presently available to treat arboviral diseases. The currently observed spread of arboviral diseases is therefore of increasing international concern [51,52]. This is particularly so, because unlike with *P. falciparum *and *P. vivax *malaria, domestic and wild animals and birds commonly serve as zoonotic reservoirs for many arboviruses that are transmitted by mosquitoes. The spread of West Nile virus (WNV) for example is associated with wild birds and *Culex *mosquitoes in Europe [53]. Hence the increased breeding of salinity-tolerant arboviral vectors, e.g. *Cx tarsalis *[38], or the adaptation of freshwater arboviral vectors of high vectorial capacity, e.g. *Ae. aegypti *and *Ae. albopictus*, to breed in brackish and saline water bodies in coastal areas can have serious health consequences worldwide.

More than half the world's population live within 60 km of a shoreline. Population density in coastal areas is expected to increase from 87 persons per km^2 ^in the year 2000 to 134 persons per km^2 ^in 2050 [54], and this trend is likely to be particularly pronounced in tropical countries where many VBD are endemic. Therefore growing numbers of people will be placed at risk by an increase in vector populations in coastal areas. Greater host density, by increasing vector-host contact, will also increase disease transmission rates. Other factors such as changing agricultural practices, irrigation development, socio-economic conditions, changes in wild and domestic animal reservoirs, and health and habitation patterns are likely to interact in a complex way with the bionomics of salinity-tolerant vectors in influencing disease transmission in coastal areas. The plethora of factors that can be involved is illustrated by a malaria outbreak in the Demerara river estuary of Guyana in the last century [[55], Additional file 2].

Brackish/saline water bodies are frequently neglected in vector control programs. For example, malaria in Sri Lanka has traditionally been endemic in the extensive coastal areas of the dry zone where large lagoons are present and inland brackish water bodies are common [33,34]. Recent evidence suggests an important role for salinity-tolerant *An. sundaicus *s.l. in transmitting coastal malaria [22]. However, larval vector control measures in the country have until now focused on inland freshwater bodies that are the preferred breeding sites for the principal vector, *An. culicifacies *species E [34]. Attention has also been drawn to the need for treating brackish water breeding sites of *Verrallina funerea*, a mosquito vector of arboviruses, in eastern Australia [56].

### Effective countermeasures can mitigate the effect of rising sea levels on increasing the transmission of vector-borne infectious diseases

Effective malaria control measures employed over the past few decades in many countries has amply compensated for any tendency to increase disease transmission due to climate change over this period [57]. Similarly, appropriate control measures can prevent increased transmission of VBD due to the effects of rising sea levels. Low-resolution maps of the global distribution of the dominant anopheline vectors of malaria, including salinity-tolerant mosquito species, are now available [58]. Similar maps need to be developed for other disease vectors. Large projects are also underway to map the impact of rising sea levels on coasts [14]. The findings can be used in conjunction with studies modelling the effects of climate change to arrive at an overall risk map for VBD. This information together with advances in understanding the biology of the relevant vectors and pathogens will be useful for developing effective counter measures. Examples of engineering measures that can be applied include steps to reduce the development of new coastal swamps and other potential brackish/saline water breeding sites, and tidal flows in estuaries. Greater funding for the relevant biological research, detailed surveillance of disease transmission and vector densities and the application of vector control measures to brackish/saline water habitats are other measures that can be readily initiated at national and international levels. It is also important that all national agencies responsible for the different concerned sectors e.g. health, agriculture, coastal planning, environment, irrigation, livestock development, etc. are made aware of the increased risk of VBD in coastal areas in order to incorporate it into their strategic plans.

## Competing interests

The authors declare that they have no competing interests.

## Authors' contributions

RR wrote the article and SNS contributed to its content. Both authors read and approved the final manuscript.

## Authors' information

RR (MA, PhD Cantab) is a Professor in the Institute of Health Sciences, Universiti Brunei Darussalam and a Visiting Researcher in the University of Cambridge with interests in Malaria, Vector-borne Diseases and Sustainable Development. SNS (BSc, PhD Colombo) is a Senior Lecturer in the Department of Zoology, University of Jaffna with an interest in Medical Entomology.

## Pre-publication history

The pre-publication history for this paper can be accessed here:

http://www.biomedcentral.com/1471-2334/11/18/prepub

## Supplementary Material

Additional File 1 **Common salinity-tolerant mosquito vectors of human disease**. This file contains a table of important mosquito vector species that breed in brackish and saline waters, their geographical distribution and the major pathogens that they transmit. Click here for file

Additional File 2 **Illustrative case studies**. This file contains summaries of case studies from different countries illustrating aspects of the hypothesis. Click here for file
