# Supplementary material for: Possible impact of rising sea levels on vector-borne infectious diseases
Source: BMC Infect Dis. 2011 Jan 18;11:18. doi: 10.1186/1471-2334-11-18 (PMC3035583; doi:10.1186/1471-2334-11-18)
Supplement: Additional File 1 — Common salinity-tolerant mosquito vectors of human disease. This file contains a table of important mosquito vector species that breed in brackish and saline waters, their geographical distribution and the major pathogens that they transmit. [file 1471-2334-11-18-S1.DOC]

**Additional File 1 - Common salinity-tolerant mosquito vectors of human disease**

Important mosquito vector species that breed in brackish and saline habitats, their geographical distribution and the major pathogens that they transmit are presented in this Table. The data was abstracted from Reference 17 cited in the article.

| **Species** | **Distribution** | **Known Transmitted Pathogens** |
| --- | --- | --- |
| *Aedes dorsalis* | Pacific coast of  N America | West Nile virus and Western equine encephalitis virus |
| *Ae. (Ochlerotatus) taeniorhynchus* | N & S America | Eastern equine encephalitis virus |
| *Ae. togoi* | North Pacific rim | Japanese encephalitis virus and filarial parasites |
| *Ae. (Ochlerotatus) vigilax* | Australasia, SE Asia | Filarial parasites, Ross River virus, Barmah forest virus |
| *Anopheles albimanus & An. aquasalis* | N & S America, Caribbean | Malaria parasites |
| *An. atroparvus* | Coast of W Europe | Malaria parasites |
| *An. farauti & An.annulipes* | Australasia | Malaria parasites |
| *An. melas and An. merus* | Africa | Malaria parasites |
| *An. multicolor* | N Africa, Middle East | Malaria parasites |
| *An. sacharovi* | Russia, S Europe | Malaria parasites |
| *An. subpictus* | Asia | Malaria and filarial parasites |
| *An. sundaicus* | S Asia, SE Asia, China | Malaria parasites |
| *Culex sitiens* | Indian ocean rim countries | Japanese encephalitis virus and Ross River virus |
| *Cx. tritaeniorhyncus* | Russia, Middle East, Africa, India | Japanese encephalitis virus |
