# Supplementary material for: Possible impact of rising sea levels on vector-borne infectious diseases
Source: BMC Infect Dis. 2011 Jan 18;11:18. doi: 10.1186/1471-2334-11-18 (PMC3035583; doi:10.1186/1471-2334-11-18)
Supplement: Additional File 2 — Illustrative case studies. This file contains summaries of case studies from different countries illustrating aspects of the hypothesis. [file 1471-2334-11-18-S2.DOC]

**Additional File 2 – Illustrative case studies**

Case studies from different countries illustrating aspects of the hypothesis are summarised in this file.

**2.1 Increase in the density of *Aedes* *camptorhynchus* due to greater salinisation of inland fresh water bodies as a result of intensive agriculture in Western Australia**

*Aedes* *camptorhynchus* is a vector of Ross River virus (RRV) in Australia. Larvae of *Ae.* *camptorhynchus* are commonly able to develop into adults in brackish and hypersaline waters (<62ppt salt) in both inland and coastal areas of Western Australia [Reference 28 in article]. Higher densities of *Ae.* *camptorhynchus* have been associated with increasing salinisation of freshwater bodies due to intensive wheat farming in southwest Western Australia [References 26-29 in article]. Increased salinity is postulated to reduce biotic regulation of *Ae*. *camptorhynchus* larvae further contributing to higher vector density [Reference 29 in article]. Low human population density, vertebrate host distribution and seasonal mosquito population dynamics may however have limited the incidence of human RRV disease in the area [References 26 and 27 in article].

**2.2 High local density of a malaria vector can help spread malaria elsewhere**

The potential for prolific localised breeding of a relatively inefficient malaria vector to trigger widespread malaria epidemics is illustrated with an example from Sri Lanka. The Mahaweli project in North Central Sri Lanka was an agricultural development in the 1980s that led to the extensive conversion of dry zone forest to agricultural land irrigated by a network of canals drawing water from the island’s longest river – the Mahaweli River. *An. annularis* was shown to breed preferentially in the newly constructed irrigation canals in the Weheragala locality where there was a high prevalence of malaria [Reference 47 in article]. Although *An. annularis* showed lower sporozoite rates than *An. culicifacies* in Weheragala, it became the predominant local vector with very high entomological inoculation rates due primarily to its high density in the area [Reference 47 in article]. The resultant local focus of intense malaria transmission is postulated to have been amplified and spread elsewhere in the country by the more ubiquitous and efficient *An. culicifacies* vector resulting in frequent epidemics of malaria during this period.

**2.3 Interactions between factors such as changes in vector physiology and abundance, human settlement, agricultural practice and livestock numbers in altering transmission of malaria in the Demerara river estuary in Guyana**

*An. darlingi*, an anthropophagic and endophilic freshwater species and the predominant malaria vector in the Demerara river estuary in Guyana, was eliminated, together with malaria, in the estuary by an indoor DDT spraying campaign in 1946-1950. The salinity-tolerant, zoophagic and exophilic *An. aquasalis* was a minor vector in the area, although it was a well-established malaria vector in the Caribbean islands and elsewhere along the Northeast coast of South America [Reference 17 in article]. It had traditionally increased in numbers during dry seasons due to saline water intrusion in the estuary. The elimination of *An. darlingi* in the area was accompanied by the conversion of pastures into rice fields, and increased human settlement. An outbreak of *P*. *vivax* malaria that occurred in 1960-61 was accompanied by a marked increase in *An. aquasalis* collection indoors. It was concluded that an increase in the proportion of humans relative to large animal livestock in the area had caused *An. aquasalis* to adapt to become more anthrophagic and endophilic and re-establish endogenous malaria transmission in the area [Reference 55 in article].
